# Expression signature, prognosis value and immune characteristics of cathepsin F in non-small cell lung cancer identified by bioinformatics assessment

**DOI:** 10.1186/s12890-021-01796-w

**Published:** 2021-12-20

**Authors:** Liyuan Song, Xianhui Wang, Wang Cheng, Yi Wu, Min Liu, Ruizi Liu, Shenyi Zhang, Hong Xia, Hao Liu, Xuejiao Tai, Huzi Zhao, Xihua Li, Fuyun Ji

**Affiliations:** 1 Department of Medical Biology, School of Basic Medical Science, Hubei University of Medicine, Shiyan, 442000 Hubei Province China; 2 Institute of Biomedical Research, Hubei University of Medicine, Shiyan, 442000 Hubei Province China; 3 Taihe Hospital, Hubei University of Medicine, Shiyan, 442000 Hubei Province China; 4 School of Clinical Medicine, Shandong First Medical University, Jinan, 250000 Shandong Province China; 5 Department of Pathology, School of Basic Medical Science, Hubei University of Medicine, Shiyan, 442000 Hubei Province China; 6 Hubei Key Laboratory of Embryonic Stem Cell Research, School of Basic Medical Science, Hubei University of Medicine, Shiyan, 442000 Hubei Province China

**Keywords:** CTSF, NSCLC, Prognosis, Immune response, Immunotherapy

## Abstract

**Background:**

In recent years, immunotherapies and targeted therapies contribute to population-level improvement in NSCLC cancer-specific survival, however, the two novel therapeutic options have mainly benefit patients containing mutated driven genes. Thus, to explore other potential genes related with immunity or targeted therapies may provide novel options to improve survival of lung cancer patients without mutated driven genes. CTSF is unique in human cysteine proteinases. Presently, CTSF has been detected in several cell lines of lung cancer, but its role in progression and prognosis of lung cancer remains unclear.

**Methods:**

CTSF expression and clinical datasets of lung cancer patients were obtained from GTEx, TIMER, CCLE, THPA, and TCGA, respectively. Association of CTSF expression with clinicopathological parameters and prognosis of lung cancer patients was analyzed using UALCAN and Kaplan–Meier Plotter, respectively. LinkedOmics were used to analyze correlation between CTSF and CTSF co-expressed genes. Protein–protein interaction and gene–gene interaction were analyzed using STRING and GeneMANIA, respectively. Association of CTSF with molecular markers of immune cells and immunomodulators was analyzed with Immunedeconv and TISIDB, respectively.

**Results:**

CTSF expression was currently only available for patients with NSCLC. Compared to normal tissues, CTSF was downregulated in NSCLC samples and high expressed CTSF was correlated with favorable prognosis of NSCLC. Additionally, CTSF expression was correlated with that of immune cell molecular markers and immunomodulators both in LUAD and LUSC. Noticeably, high expression of CTSF-related CTLA-4 was found to be associated with better OS of LUAD patients. Increased expression of CTSF-related LAG-3 was related with poor prognosis of LUAD patients while there was no association between CTSF-related PD-1/PD-L1 and prognosis of LUAD patients. Moreover, increased expression of CTSF-related CD27 was related with poor prognosis of LUAD patients while favorable prognosis of LUSC patients.

**Conclusions:**

CTSF might play an anti-tumor effect via regulating immune response of NSCLC.

**Supplementary Information:**

The online version contains supplementary material available at 10.1186/s12890-021-01796-w.

## Background

Lung cancer is the most frequently occurring cancer and the leading cause of cancer death worldwide with an approximated 2.2 million new cancer cases and 1.8 million deaths in 2020 [1]. It is estimated that number of lung cancer deaths will increase to 3 million in 2035 [2, 3]. Non-small cell lung cancer (NSCLC) and small cell lung cancer (SCLC) are the two major histologic subtypes of lung cancer, and the former accounts for about 85% of all lung cancer cases and are mainly composed of lung adenocarcinoma (LUAD) and lung squamous cell carcinoma (LUSC). Prognosis for lung cancer is very poor. Five-year survival rates of lung cancer vary from 4–17% depending on subtypes and stage at the time of its diagnosis [4]. In recent years, immunotherapies and targeted therapies contribute to population-level improvement in NSCLC cancer-specific survival, however, the two novel therapeutic options have been mainly beneficial for those who contain the mutated driven genes, such as genes encoding PD-1/PD-L1 [5], anaplastic lymphoma kinase (ALK) [6], or epidermal growth factor receptor (EGFR) [7]. Thus, to explore other potential genes related with immunity or targeted therapies may provide novel options to improve survival of lung cancer patients who are not suitable for the current immunotherapies or targeted therapies.

Cysteine proteinases are a widespread group of enzymes that catalyze hydrolysis of many different proteins and play a major role in intracellular protein degradation and turnover [8, 9]. These proteolytic enzymes can be subdivided into more than 20 different families, and papain family is the largest one [10]. Human cysteine proteinases of papain family are main component of lysosomal protein hydrolysis system and contain 15 different family members, including cathepsin A-H, K, L, O, S, V, W, and Z [11]. These cathepsins (CTSs) contain a series of conserved features in their active site and are synthesized as preproenzymes, which are processed to corresponding proenzymes and targeted to lysosomes by mannose 6-phosphate signal attached to them [12]. Among these cysteine proteinases, lysosomal CTSB, CTSD, CTSL, and CTSS have been shown to be involved in tumor malignant progression and are potential targets of anti-tumor therapy [13–16]. Cloned from a human prostate cDNA library and unique in cathepsin because of having an extended N-terminal anterior region containing a cystatin domain, CTSF gene encodes a polypeptide of 484 amino acids with the same domain organization as other cysteine proteinases. The wide expression of CTSF in human tissues suggests that CTSF may be involved in protein catabolism [12]. Additionally, CTSF likely plays a regulatory role in processing invariant chain which is associated with major histocompatibility complex (MHC) class II [17, 18]. In fish, CTSF appears to take part in innate immune responses [19]. Presently, CTSF has been detected in some human cancer cell lines (such as HL-60, HeLa, K-562, MOLT-4, Raji, SW480, A549 and G361) [12], however, its role in progression and prognosis of tumors is still unclear. In the study, CTSF expression in NSCLC were analyzed using the public databases online available. The bioinformatics assessment revealed that CTSF may play anticancer effect in NSCLC by regulating immune responses.

## Materials and methods

### CTSF expression in human cancers and cancer cell lines

CTSF expression in human normal tissues and Pan-cancerous tumors were obtained from Genotype-tissue expression (GTEx) and Tumor Immune Estimation Resource (TIMER1.0), respectively. GTEx provides RNA-seq data from 53 normal tissues across nearly 1000 people (http://xena.ucsc.edu/). TIMER is a comprehensive resource for systematical analysis of immune infiltrates across diverse cancer types (https://cistrome.shinyapps.io/timer/). CTSF expression in human normal tissues and Pan-cancers were analyzed using web browser-basis tools (https://gtexportal.org/home/gene/ENSG00000174080 and https://cistrome.shinyapps.io/timer/, respectively). Expression of CTSF in human cancer cell lines was downloaded from Cancer Cell Line Encyclopedia (CCLE) (https://www.betastasis.com/tissues/cancer_cell_line_encyclopedia/gene_expression_barplot/).

### Association of CTSF expression with prognosis of NSCLC

Expression of CTSF in LUAD and LUSC was derived from The Cancer Genome Atlas (TCGA) dataset (https://www.cancer.gov/about-nci/organization/ccg/research/structural-genomics/tcga). TCGA has generated large amounts of Next-generation sequencing (NGS) data with a landscape of more than 11,000 tumors across 33 cancer types until 2018. Unpaired Wilcoxon test was used to evaluate distributions of CTSF expression in unpaired normal and tumor tissues of TCGA-LUAD and TCGA-LUSC cohorts, and paired Wilcoxon test was used to analyze CTSF expression between paired tumors and corresponding adjacent normal tissues of TCGA-LUAD and TCGA-LUSC cohorts. UALCAN was used to explore association of CTSF expression with clinicopathological parameters of LUAD and LUSC patients, including gender, age, smoking habits, stage, metastasis, TP53 mutation statuses and race (http://ualcan.path.uab.edu/cgi-bin/TCGAExResultNew2.pl?genenam=CTSF&ctype=LUAD, and http://ualcan.path.uab.edu/cgi-bin/TCGAExResultNew2.pl?genenam=CTSF&ctype=LUSC, respectively). UALCAN allows analysis of relative expression of a query gene(s) across tumor and normal samples, as well as in various tumor sub-groups based on individual cancer stages, tumor grade or other clinicopathological features. One-way ANOVA was used to perform a comparison for continuous variables among groups ≥ 3 on web browser-basis analysis. *P* < 0.05 was considered statistically significant [20]. Immunohistochemistry (IHC) staining and subcellular localization of CTSF were derived from The Human Protein Atlas (THPA) dataset (https://www.proteinatlas.org/ENSG00000174080-CTSF/pathology/lung+cancer). THPA integrates RNA and protein expression data corresponding to ∼80% of human protein-coding genes with access to the primary data for both RNA and protein analysis on an individual gene level. Currently, CTSF expression in GTEx, TCGA and THPA databases are only available for LUAD and LUSC.

Kaplan–Meier Plotter was used to analyze CTSF expression with prognosis of NSCLC patients (http://kmplot.com/analysis/index.php?p=service&cancer=lung). Kaplan–Meier plotter can assess effect of 54,675 genes on survival of cancer patients with 10,461 cancer samples. Prognostic parameters included overall survival (OS), first-progression survival (FPS), and post-progression survival (PPS). FPS refers to the duration of disease progression from first-line treatment to first progression. PPS refers to the duration of disease progression from first-line treatment to death. Since post-progression therapy influences OS, PPS is of interest as a determinant of OS and considered as the difference between median OS and median progression-free survival (PFS) or time to tumor progression [21, 22]. Patients with LUAD and LUSC were divided into two subgroups based on median expression of CTSF (high vs. low expression), respectively. Survival diagram, Hazard ratio (HR) with 95% confidence intervals (95% CIs) and logrank *P* value were calculated and plotted in R using the “survplot” function of the “survival” Bioconductor package as described [23].

Additionally, TCGA-LUAD and TCGA-LUSC database were used to analyze correlations of CTSF expression with tumor mutational burden (TMB) and microsatellite instability (MSI) in LUAD and LUSC, respectively. Spearman’s correlation analysis was used to describe the correlation between quantitative variables without a normal distribution (https://www.aclbi.com/static/index.html#/tcga). A *p *value of less than 0.05 was considered statistically significant.

### Genes co-expressed with CTSF in NSCLC

LinkFinder module and LinkInterpreter module of LinkedOmics were used to analyze correlation of CTSF with other genes co-expressed with CTSF in LUAD and LUSC, respectively. LinkedOmics database is a Web-based platform for analyzing 32 TCGA cancer-associated multi-dimensional datasets (http://www.linkedomics.org/admin.php) [24]. All results were graphically presented in volcano plots, heat maps or scatter plots. Co-expressed genes with CTSF were categorized using Gene Ontology (GO) and Kyoto Encyclopedia of Genes and Genomes (KEGG) enrichment analysis (http://www.linkedomics.org/admin.php).

Moreover, protein–protein interaction (PPI) network and gene–gene interaction network for CTSF were constructed using STRING and GeneMANIA, respectively. STRING is a flexible and user-friendly database of known and predicted PPIs, covering 24,584,628 proteins from 5,090 organisms currently (https://www.string-db.org/cgi/network?taskId=bbIwHMDa6Z9y&sessionId=bISFiNqXFgX5). Interactions in STRING include direct (physical) and indirect (functional) associations, stemming from computational prediction, knowledge transfer between organisms, and interactions aggregated from other (primary) databases. In the study, PPI network was constructed by setting medium confidence at 0.400. GeneMANIA generates a list of genes with similar functions to the query gene and constructs an interactive functional-association network using a very large set of functional association data (http://genemania.org/search/homo-sapiens/CTSF). Association data include protein and genetic interactions, pathways, co-expression, co-localization and protein domain similarity. GeneMANIA can also be used to find new members of a pathway or complex, additional genes missed in the query input list or new genes with a specific function, such as protein kinases. In the present study, GeneMANIA was used to construct a gene–gene interaction network for CTSF to evaluate the potential functions of these genes. Each node represents a gene. The node color represents the possible functions of each gene.

### CTSF and molecular markers of immune cells in NSCLC

Association of CTSF with molecular markers of immune cells between NSCLC and normal tissues was analyzed with Immunedeconv, an R package which integrates six state-of-the-art algorithms, including TIMER, xCell, MCP-counter, CIBERSORT, EPIC and quanTIseq (https://www.aclbi.com/static/index.html#/immunoassay). TIMER was utilized to analyze correlation of copy numbers of CTSF gene with infiltration levels of immune cells using a two-sided Wilcoxon rank-sum test.

CTSF-related immunomodulators were analyzed with TISIDB to elucidate tumor-immune system interactions (http://cis.hku.hk/TISIDB/browse.php?gene=CTSF). TISIDB is a website for gene- and tumor-immune interaction, built based on data collected and integrated from following resources: PubMed database, high-throughput screening data investigating the responses of tumor cells to T cytotoxic cells, exome and RNA sequencing data of patients receiving immunotherapy, TCGA, and other public databases. Immunoinhibitors and immunostimulators that were significantly correlated with CTSF expression were chosen based on *p* < 0.05 (Spearman correlation test). Association of immunomodulators with prognosis of LUAD and LUSC patients was analyzed using Kaplan–Meier plotter as described above.

## Results

### CTSF was downregulated significantly in most of human cancer tissues

**Fig. 1:**
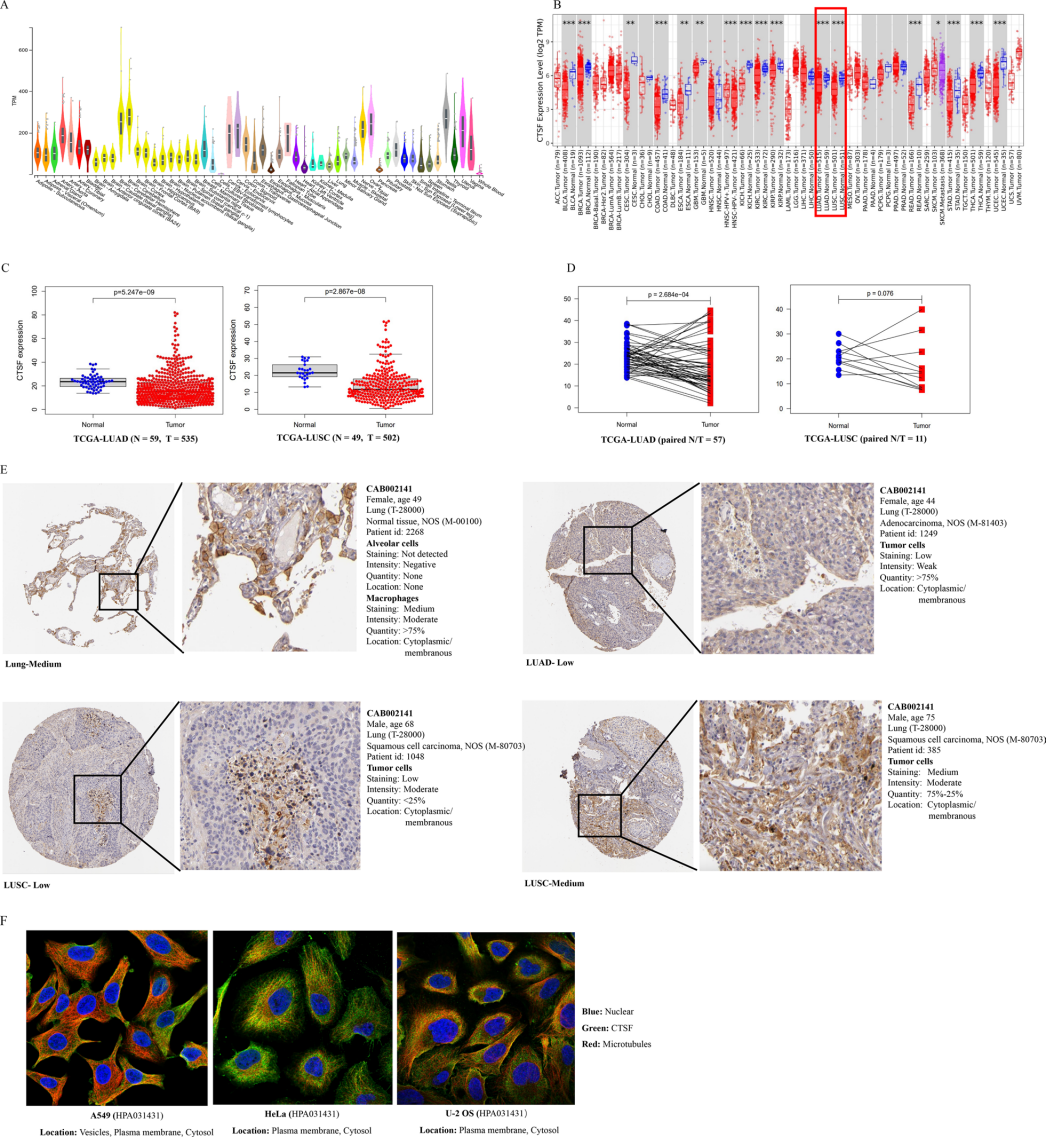
CTSF Expression in human normal tissues and cancer samples. **A** CTSF expression in human normal tissues (GTEx database); **B** Comparison of CTSF expression between human normal tissues and cancer tissues (TIMER database); **C** Comparison of CTSF expression between unpaired normal lung tissues and tumor tissues of LUAD (left) and LUSC (right), respectively (TCGA database); **D** comparison of CTSF expression between paired tumor tissues and corresponding paracancerous tissues of LUAD (left) and LUSC (right), respectively (TCGA database); **E** IHC staining of CTSF in normal lung tissues and cancer samples of patients with LUAD or LUSC, respectively (THPA database); **F** subcellular localization of CTSF in different cell lines of human cancers (THPA database)

CTSF was detected in almost all of human normal tissues. Relatively, higher expressed CTSF was observed in Artery-Aorta, Brain (cerebellar hemisphere and cerebellum), Cervix (ectocervix and endocervix), Fallopian tube, Nerve (tibial), ovary, Testis and Uterus (GTEx dataset, TPM > 200, Fig. 1A). Exploration of CTSF expression in pan-cancers using TIMER revealed that compared to normal tissues, CTSF was downregulated significantly in most of cancer tissues, such as Bladder urothelial carcinoma (BLCA), Breast invasive carcinoma (BRCA), Cervical squamous cell carcinoma and endocervical adenocarcinoma (CESC), Cholangio carcinoma (COAD), Esophageal carcinoma (ESCA), Glioblastoma multiforme (GBM), Kidney chromophobe (KICH), Kidney renal clear cell carcinoma (KIRC), Kidney renal papillary cell carcinoma (KIRP), Lung adenocarcinoma (LUAD), Lung squamous cell carcinoma (LUSC), Rectum adenocarcinoma (READ), Stomach adenocarcinoma (STAD), Thyroid carcinoma (THCA), and Uterine corpus endometrial carcinoma (UCEC) (Fig. 1B). CTSF was detected in many human cancer cell lines including lung cancer (CCLE dataset, Additional file 1: Table S1).

**Table 1:** Correlation between CTSF expression and that of immune cell markers using TIMER

Description	Gene markers	LUAD (n = 515)				LUSC (n = 501)			
		None		Purity		None		Purity	
		cor	*p*	cor	*p*	cor	***p***	cor	***p***
CD8^+^ T cell	CD8A	− 0.075	0.088	− 0.092	*	0.104	*	0.101	*
	CD8B	− 0.033	0.948	− 0.015	0.739	0.229	***	0.220	***
T cell (general)	CD3D	− 0.016	0.709	− 0.020	0.651	0.076	0.090	0.068	0.139
	CD3E	0.032	0.473	0.042	0.353	0.118	**	0.118	**
	CD2	0.020	0.648	0.025	0.577	0.107	*	0.102	*
B cell	CD19	0.139	**	0.176	***	0.115	*	0.115	*
	CD79A	0.157	***	0.191	***	0.154	***	0.168	***
Monocyte	CD86	0.039	0.372	0.004	0.388	0.130	*	0.125	0.006
	CD115(CSF1R)	0.133	**	0.145	**	0.186	***	0.197	***
TAM	CCL2	0.085	0.053	0.094	*	0.215	***	0.211	***
	CD68	0.008	0.860	0.013	0.776	0.052	0.245	0.038	0.413
	IL10	0.093	*	0.112	*	0.186	***	0.190	***
M1 Macrophage	INOS(NOS2)	0.096	*	0.109	*	0.135	**	0.121	**
	IRF5	0.025	0.564	0.031	0.492	0.015	0.740	0.009	0.840
	COX2(PTGS2)	0.046	0.295	0.037	0.407	0.103	*	0.095	*
M2 Macrophage	CD163	0.012	0.794	0.009	0.843	0.135	**	0.130	**
	VSIG4	0.024	0.592	0.032	0.480	0.140	**	0.134	**
	MS4A4A	0.041	0.355	0.050	0.263	0.118	**	0.110	*
Neutrophils	CD66B(CEACAM8)	0.187	***	0.202	***	0.130	**	0.109	*
	CD11B(ITGAM)	0.100	*	0.109	*	0.163	***	0.164	***
	CCR7	0.118	**	0.152	***	0.153	***	0.153	***
Natural killer cell	KIR2DL1	− 0.083	0.060	− 0.095	*	0.030	0.051	0.021	0.652
	KIR2DL3	− 0.145	***	− 0.143	**	0.019	0.677	0.012	0.796
	KIR2DL4	− 0.213	***	− 0.236	***	0.001	0.986	− 0.010	0.890
	KIR3DL1	− 0.081	0.066	− 0.086	0.556	0.103	*	0.087	0.058
	KIR3DL2	− 0.108	*	− 0.121	**	− 0.021	0.634	− 0.034	0.465
	KIR3DL3	− 0.151	***	− 0.154	***	0.040	0.377	0.036	0.434
	KIR2DS4	− 0.071	0.108	− 0.073	0.105	0.027	0.550	0.024	0.600
Dendritic cell	HLA-DPB1	0.186	***	0.208	***	0.156	***	0.168	***
	HLA-DQB1	0.134	**	0.155	***	0.095	*	0.098	*
	HLA-DRA	0.156	***	0.173	***	0.140	**	0.145	**
	HLA-DPA1	0.179	***	0.197	***	0.151	***	0.157	***
	BDCA-1(CD1C)	0.197	***	0.217	***	0.101	*	0.103	*
	BDCA-4(NRP1)	0.061	0.168	0.059	0.190	0.262	***	0.184	***
	CD11c(ITGAX)	0.056	0.203	0.059	0.191	0.088	*	0.094	*
Th1	T-bet(TBX21)	− 0.029	0.514	− 0.026	0.566	0.078	0.082	0.078	0.090
	STAT4	0.051	0.248	0.056	0.213	0.189	***	0.211	***
	STAT1	− 0.121	**	− 0.144	**	0.031	0.495	0.026	0.569
	IFN-γ (IFNG)	− 0.206	***	− 0.225	***	− 0.051	0.256	− 0.034	0.462
	TNF-α (TNF)	0.048	0.274	0.061	0.173	0.075	0.092	0.067	0.142
Th2	GATA3	0.071	0.106	0.075	0.097	0.310	***	0.307	***
	STAT6	0.102	**	0.113	*	0.043	0.340	0.042	0.358
	STAT5A	0.167	***	0.183	***	0.245	***	0.259	***
	IL13	0.024	0.588	0.024	0.598	0.033	0.466	0.020	0.656
Tfh	BCL6	0.188	***	0.192	***	− 0.031	0.486	− 0.034	0.458
	IL21	− 0.071	0.108	− 0.071	0.115	− 0.044	0.323	− 0.051	0.264
Th17	STAT3	0.206	***	0.213	***	0.189	***	0.187	***
	IL17A	− 0.132	**	− 0.126	**	− 0.128	**	− 0.119	**
Treg	FOXP3	0.050	0.262	0.059	0.189	0.168	***	0.176	***
	CCR8	0.023	0.596	0.018	0.696	0.151	***	0.142	**
	STAT5B	0.286	***	0.293	***	0.287	***	0.289	***
	TGFβ (TGFB1)	0.171	***	0.181	***	0.084	0.059	0.075	0.102
T cell exhaustion	PD-1 (PDCD1)	0.002	0.958	0.001	0.991	0.137	**	0.146	**
	CTLA4	− 0.039	0.378	− 0.048	0.289	0.079	0.078	0.074	0.107
	LAG3	− 0.080	0.069	− 0.098	*	0.057	0.201	0.051	0.264
	TIM-3 (HAVCR2)	0.002	0.969	0.001	0.984	0.093	*	0.085	0.065
	GZMB	− 0.239	***	− 0.273	***	0.009	0.848	− 0.006	0.901

### High CTSF expression was correlated with favorable prognosis of NSCLC

As shown in Fig. 1C, unpaired Wilcoxon test demonstrated that CTSF expression was markedly downregulated both in LUAD (left) and LUSC (right) tissues compared to that of normal tissues. Paired Wilcoxon test revealed that CTSF expression was higher in some tumor tissues while lower in the other tumor tissues compared with that of the corresponding paracancerous tissues (Fig. 1D; left: LUAD; right: LUSC). Interestingly, IHC staining of CTSF showed that CTSF was not detected in alveolar cells while medium staining of CTSF was observed in macrophage cells in normal lung tissues (Fig. 1E). Moreover, stronger IHC staining of CTSF was detected in infiltrating immune cells such as macrophage cells than that of cancerous cells both in LUAD and LUSC tissues (Fig. 1E). As shown in Fig. 1F, CTSF was localized to plasma membrane, cytosol, and vesicles in tumor cells.

**Fig. 2:**
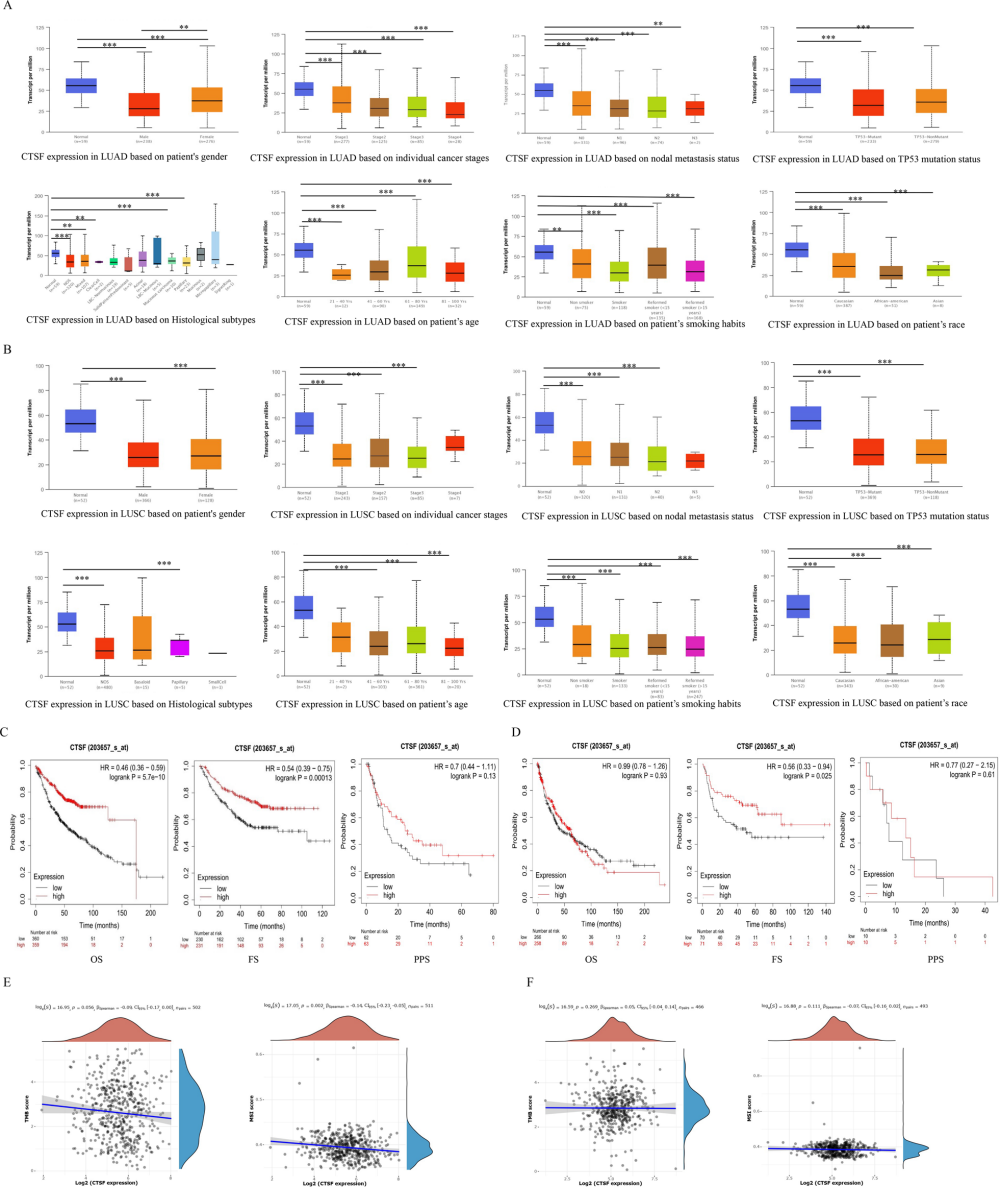
Associations of CTSF expression with clinical characteristics of NSCLC. **A**, **B** Associations of CTSF expression with clinical characteristics of LUAD and LUSC, respectively; **C**, **D** associations of CTSF expression with prognosis of LUAD (**C**) and LUSC (**D**), respectively; **E**, **F** Correlations of CTSF expression with TMB and MSI in LUAD (**E**) and LUSC (**F**), respectively

Analysis of CTSF expression with clinical characteristics of LUAD showed that CTSF expression was significantly associated with gender, age, tumor stage, lymph node metastasis, smoking habits, histological subtypes, TP53-muation status, and race, respectively. CTSF expression decreased with stage, metastasis and smoking in LUAD (Fig. 2A). Similar findings were obtained in LUSC (Fig. 2B). Kaplan–Meier survival analysis demonstrated that high CTSF expression was correlated with favorable prognosis of LUAD (OS: HR = 0.46, *p* = 5.7e−10; FS: HR = 0.54, *p* = 1.3e−04) (Fig. 2C). In LUSC, high expressed CTSF was found to be significantly associated with better FS (HR = 0.56, *p* = 0.025) (Fig. 2D).

Analysis of CTSF expression with TMB and MSI demonstrated that CTSF expression was negatively related with MSI (*p* = 0.002), while no significant association was found between CTSF expression and TMB in LUAD (Fig. 2E). There was no significant association between CTSF expression and TMB or MSI in LUSC (Fig. 2F).

### CTSF might play an important role in antigen presentation for immune response of NSCLC

**Fig. 3:**
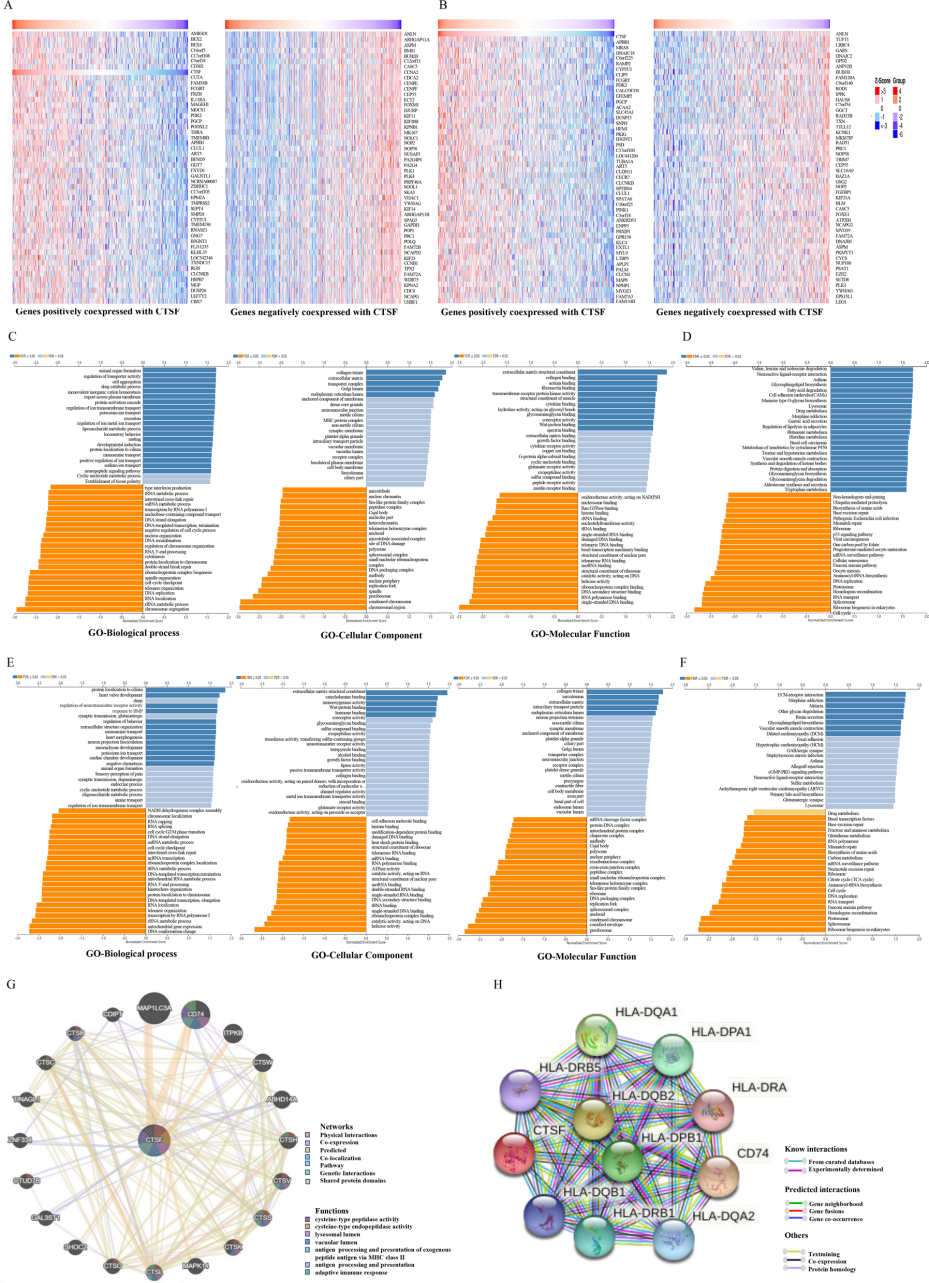
Genes Co-expressed with CTSF in NSCLC (LinkedOmics). **A**, **B** Heatmaps showing the top 50 genes positively (left) or negatively (right) co-expressed genes with CTSF in LUAD (**A**) and LUSC (**B**), respectively; **C**, **D** enriched genes co-expressed with CTSF in LUAD using GO (**C**) and KEGG (**D**) analysis, respectively; **E**, **F** enriched genes co-expressed with CTSF in LUSC using GO (**E**) and KEGG (**F**) analysis, respectively; **G** Gene–gene interaction network constructed using GeneMANIA; (H) PPI network constructed by STRING (medium confidence = 0.400)

To better understand biological implications of CTSF in NSCLC, “LinkFinder” module in LinkedOmics was applied to explore genes co-expressed with CTSF. As plotted in Additional file 2: Fig. S1A, expression of 4,452 genes (red dots) was positively correlated with that of CTSF, while expression of 2,041 genes (green dots) was negatively correlated with that of CTSF in LUAD (FDR < 0.01). In LUSC, expression of 5,019 genes (red dots) was positively correlated with CTSF expression, while expression of 1,820 genes (green dots) was negatively correlated with CTSF expression (FDR < 0.01, Additional file 2: Fig. S1B). Heatmaps of the top 50 genes positively or negatively associated with CTSF in LUAD and LUSC were shown in Fig. 3A and B, respectively. GO term annotation showed that molecular function of genes co-expressed positively with CTSF in LUAD were mainly involved in collagen trimer, extracellular matrix, transport complex, Golgi limen, and endoplasmic reticulum lumen. In contrast, molecular function of genes co-expressed negatively with CTSF were mainly involved in chromosomal region, condensed chromosome, preribosome, spindle, replication fork and others (GO-Molecular Function, Fig. 3C). KEGG analysis demonstrated genes co-expressed positively with CTSF in LUAD were primarily enriched in Valine, leucine and isoleucine degradation, Asthma and Cell adhesion molecules (CAMs), while genes co-expressed negatively with CTSF in LUAD were primarily enriched in Cell cycle, ribosome biogenesis in eukaryotes, spliceosome, RNA transport, proteasome and others (Fig. 3D). GO term annotation displayed that molecular function of genes co-expressed positively with CTSF in LUSC were mainly involved in extracellular matrix structure constituent, collagen binding, actinin binding, fibronectin binding, transmembrane receptor protein kinase activity, structure constituent of muscle, hydrolase activity, acting on glycosyl bonds, glycosaminoglycan binding, coreceptor activity and Wnt-protein binding. In contrast, molecular function of genes co-expressed negatively with CTSF in LUSC were mainly involved in single-stranded DNA binding, RNA polymerase binding, DNA secondary structure binding and others (Fig. 3E). KEGG analysis showed genes co-expressed positively with CTSF in LUSC were primarily enriched in ECM-receptor interaction, Morphine addiction, Malaria, Other glycan degradation, Renin secretion, Glycosphingolipid biosynthesis, Vascular smooth muscle contraction, and Dilated cardiomyopathy (DCM), while genes co-expressed negatively with CTSF in LUSC were primarily enriched in Ribosome biogenesis in eukaryotes, Spliceosome, Proteasome and others (Fig. 3F).

Gene–gene interaction network for CTSF constructed using GeneMANIA showed that CTSF had similar function as other 20 genes (MAP1LC3A, CD74, ITPKB, CTSW, ABHD14A, CTSH, CTSV, CTSS, CTSK, MAPK14, CTSL, CTSO, SHOC2, GAL3ST1, OTUD7B, ZNF334, TINAGL1, CTSC, CTSB, and CDIPT) (Fig. 3G). Functional analysis indicated that proteins encoded by these 20 genes were significantly correlated with following terms: cysteine-type peptidase activity, cysteine-type endopeptidase activity, lysosomal lumen, vacuolar lumen, antigen processing and presentation of exogenous peptide antigen via MHC class II, antigen processing and presentation, and adaptive immune response. PPI network constructed using STRING showed that 10 proteins were interacted with CTSF (Fig. 3H). Among these proteins interacted with CTSF, HLA-DQA1, HLA-DPA1, HLA-DRA, HLA-DQA2, HLA-DRB1, HLA-DQB1, HLA-DRB5, HLA-DQB2 and HLA-DPB1 are different subunits of the human leukocyte antigen (HLA) class II, which plays a central role in immune system by presenting peptides derived from extracellular proteins. CD74 is associates with MHC and also regulates antigen presentation for immune response.

### CTSF might regulate immune infiltration of NSCLC

**Fig. 4:**
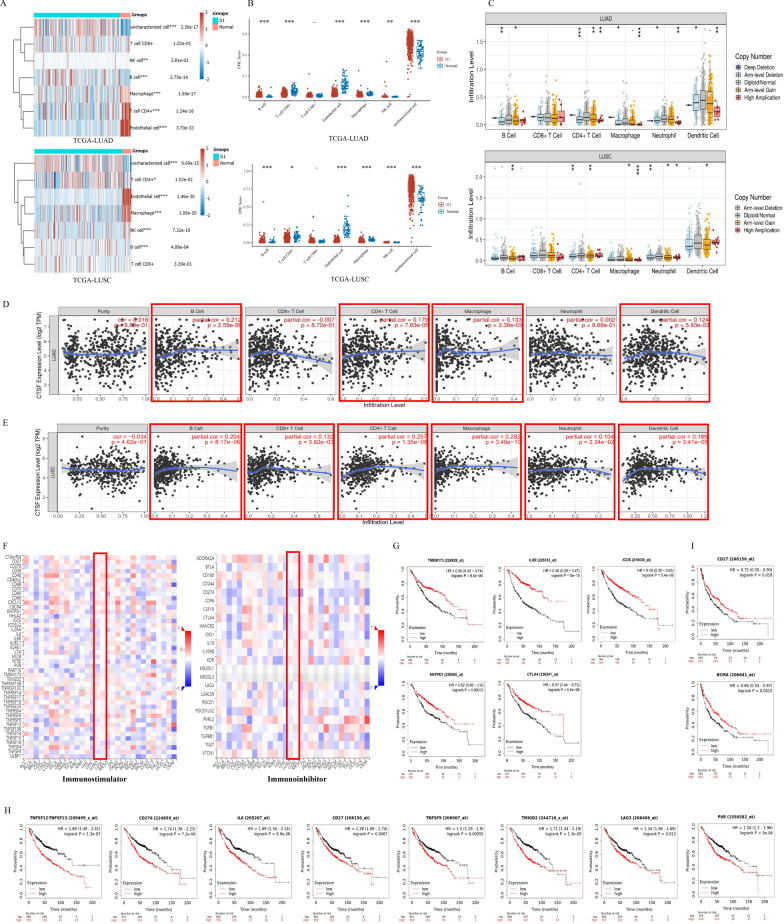
Correlation of CTSF expression with infiltration levels of immune cells and immunomodulators in NSCLC. **A** Landscape of infiltrating immune cells in cancerous and healthy biopsies for TCGA-LUAD and TCGA-LUSC cohorts, respectively; **B** EPIC scores of immune infiltrating cells between normal lung tissues and LUAD or LUSC tissues, respectively; **C** association of CTSF copy numbers with immune cell infiltration levels in LUAD (above) and LUSC (below) cohorts, respectively; **D**, **E** Correlation between CTSF expression and immune cells in LUAD (**D**) and LUSC (**E**), respectively; **F** Heatmap of CTSF-related immunomodulators in LUAD and LUSC, respectively; **G** association of CTSF-related immunostimulators (TMEM173, IL6R, ICOS, and ENTPD1) and CTSF-related immunoinhibitor (CTLA4) with favorable prognosis of LUAD patients; **H** Association of CTSF-related immunostimulators (TNFSF13, CD276, IL6, CD27, PVR, TNFSF9, TMIGD2) and CTSF-related immunoinhibitor (LAG3) with poor prognosis of LUAD patients; (I) Relationship of CTSF-related immunostimulators CD27 and TNFRSF17 (also BCMA) with good prognosis of LUSC patients

In order to explore the role of CTSF in immune response of NSCLC, TCGA-LUAD and TCGA-LUSC cohorts were downloaded to explore association of CTSF expression with infiltrating levels of immune cells. Landscape of infiltrating immune cells in cancerous and healthy biopsies for TCGA-LUAD and TCGA-LUSC cohorts was shown in Fig. 4A (above: LUAD, below: LUSC). As shown in Fig. 4A and 4B, proportions of B cells, Natural killer (NK) cells and uncharacterized cells were significantly higher while that of Macrophages, CD4^+^ T cells and Endothelial cells were significantly lower in tissues of LUAD related to normal lung tissues. In LUSC, proportions of CD4^+^ T cells, B cells, NK cells and uncharacterized cells were significantly higher while that of Macrophages and Endothelial cell were significantly lower in tumor tissues compared with that of normal tissues.

TIMER analysis demonstrated that infiltration levels of B cells, CD4^+^ T cells, Macrophages, Neutrophils, and Dendritic cells (DCs) increased with copy numbers of CTSF gene both in LUAD and LUSC (Fig. 4C). Additionally, after adjusted by tumor purity, CTSF expression was shown to be positively correlated with immune infiltration of B cells (r = 0.212, *p* = 2.59e−06), CD4^+^ T cells (r = 0.179, *p* = 7.83e−05), Macrophage (r = 0.133, *p* = 3.38e−03), and DCs (r = 0.124, *p* = 5.93e−03) in LUAD (Fig. 4D). In LUSC, besides B cells (r = 0.204, *p* = 8.17e−06), CD4^+^ T cells (r = 0.257, *p* = 1.35e−08), Macrophage (r = 0.282, *p* = 3.49e−10), and DCs (r = 0.189, *p* = 3.41e−05), immune infiltration of CD8^+^ T cells (r = 0.132, *p* = 3.82e−03) and Neutrophil (r = 0.104, *p* = 2.34e−02) were also found to be positively correlated with CTSF expression (Fig. 4E).

Additionally, the expression analysis between CTSF and molecular marker of immunological cells showed that CTSF expression was positively related with that of molecular markers of B cells, Monocytes, TAMs, M1 Macrophages, Neutrophils, DCs, Th2 cells, Tfh cells, Th17 cells and Treg cells, while negatively related with that of CD8^+^ T cells, NK cells, Th1 cells and Th17 cells in LUAD. In LUSC, CTSF expression was shown to be positively associated with that of CD8^+^ T cells, general T cells, B cells, Monocytes, TAMs, M1 macrophages, M2 macrophages, Neutrophils, DCs, Th1 cells, Th2 cells, Th17 cells and Treg cells, while negatively related with that of Th17 cells (Table 1).

### CTSF-related immunomodulators were associated with prognosis of patients with NSCLC

To further confirm association of CTSF with immune response of NSCLC, CTSF expression was analyzed with that of the known immunomodulators using TISIDB, which revealed that CTSF expression was significantly associated with that of 40 immunomodulators (including 28 immunostimulators and 12 immunoinhibitors) in LUAD or LUSC (Fig. 4F, Tables 2 and 3). Among the 12 immunoinhibitors, CTSF expression was negatively related with that of CD244 (rho = − 0.115, *p* = 8.92e−03), CD274 (also PD-L1, rho = −0.177, *p* = 5.53e−05), CTLA4 (rho= −0.140, *p* = 1.46e−03), HAVCR2 (rho= −0.087, *p* = 0.047), LAG3 (rho= −0.163, *p* = 2.03e−04), PDCD1 (also PD-1, rho= −0.089, *p* = 0.044), PVRL2 (rho= −0.102, *p* = 0.020), and TIGIT (rho= −0.151, *p* = 5.94e−04), while positively related with that of KDR (rho = 0.131, *p* = 2.97e−03) and VTCN1 (rho = 0.157, *p* = 3.43e−04) in LUAD. Of the 28 immunostimulators, CTSF expression was negatively related with that of CD276 (rho= −0.088, *p* = 0.045), ICOS (rho= −0.120, *p* = 6.55e−03), IL2RA (rho= −0.137, *p* = 1.78e−03), IL6 (rho= −0.160, *p* = 2.63e−04), KLRC1 (rho= −0.181, *p* = 3.57e−05), KLRK1 (rho= −0.113, *p* = 0.010), MICB (rho= −0.165, *p* = 1.63e−04), PVR (rho= −0.263, *p* = 1.55e−09), RAETIE (rho= −0.175, *p* = 6.40e−05), TMIGD2 (rho= −0.104, *p* = 0.018), TNFRSF9 (rho= −0.141, *p* = 1.29e−03), TNFSF4 (rho= −0.090, *p* = 0.042) and TNFSF9 (rho= −0.143, *p* = 1.13e−03), while positively related with that of CD27 (rho = 0.102, *p* = 0.021), CD40LG (rho = 0.132, *p* = 2.63e−03), CXCL12 (rho = 0.167, *p* = 1.42e−04), ENTPD1 (rho = 0.101, *p* = 0.022), ICOSLG (rho = 0.106, *p* = 0.016), IL6R (rho = 0.124, *p* = 4.77e−03), TMEM173 (rho = 0.158, *p* = 3.19e−04), TNFRSF13B (rho = 0.129, *p* = 3.24e−03), TNFRSF17 (also known as BCMA, rho = 0.126, *p* = 0.004) and TNFSF13 (rho = 0.089, *p* = 0.043). In LUSC, 18 immunomodulators (including 14 immunostimulators and four immunoinhibitors) were significantly associated with CTSF expression. Of the four immunoinhibitors, CTSF expression was negatively related with that of CD274 (rho= −0.208, *p* = 2.75e−06), while positively associated with that of ADORA2A (rho = 0.174, *p* = 9.29e−05), IL10 (rho = 0.133, *p* = 2.95e−03) and KDR (rho = 0.091, *p* = 0.043). Of the 14 immunostimulators, CTSF expression was positively with that of CD27 (rho = 0.103, *p* = 0.022), CD28 (rho = 0.100, *p* = 0.025), CD40 (rho = 0.193, *p* = 1.41e−05), CD40LG (rho = 0.100, *p* = 0.026), CD70 (rho = 0.135, *p* = 2.5e−03), CXCL12 (rho = 0.150, *p* = 7.81e−04), ENTPD1 (rho = 0.175, *p* = 8.49e−05), TNFRSF14 (rho = 0.159, *p* = 3.71e−04), TNFRSF17 (rho = 0.103, *p* = 0.021), TNFRSF8 (rho = 0.090, *p* = 0.0446) and TNFSF4 (rho = 0.099, *p* = 0.0268), while negatively related with that of RAET1E (rho= −0.089, *p* = 0.046), TNFSF18 (rho= −0.154, *p* = 5.5e−04) and TNFSF9 (rho= −0.102, *p* = 0.0237) (Tables 2 and 3).

**Table 2:** Correlation of CTSF expression with that of immunoinhibitors in LUAD and LUSC by TISIDB

Immunoinhibitor	LUAD		LUSC	
	rho	*p*	rho	*p*
ADORA2A	0.058	0.188	0.174	***
BTLA	0.004	0.935	0.039	0.389
CD160	− 0.010	0.828	0.073	0.102
CD244	− 0.115	**	0.050	0.263
CD274	− 0.177	***	− 0.208	***
CD96	− 0.059	0.182	0.056	0.213
CSF1R	0.010	0.827	0.086	0.054
CTLA4	− 0.140	**	− 0.011	0.800
HAVCR2	− 0.087	*	0.033	0.463
IDO1	− 0.069	0.117	0.064	0.152
IL10	− 0.008	0.860	0.133	**
IL10RB	− 0.026	0.561	0.080	0.074
KDR	0.131	**	0.091	*
LAG3	− 0.163	***	− 0.020	0.658
LGALS9	− 0.010	0.819	− 0.043	0.339
PDCD1	− 0.089	*	0.053	0.234
PDCD1LG2	− 0.052	0.239	− 0.016	0.724
PVRL2	− 0.102	*	0.031	0.485
TGFB1	0.063	0.150	0.000	0.999
TGFBR1	− 0.056	0.201	− 0.072	0.107
TIGIT	− 0.151	***	− 0.007	0.875
VTCN1	0.157	***	0.032	0.468

**Table 3:** Correlation of CTSF expression with that of immunostimulators in LUAD and LUSC by TISIDB

Immunostimulators	LUAD		LUSC	
	rho	*p*	rho	*p*
C10orf54	0.040	0.362	0.069	0.124
CD27	0.102	*	0.103	*
CD276	− 0.088	*	0.045	0.312
CD28	0.052	0.237	0.100	*
CD40	0.026	0.551	0.193	***
CD40LG	0.132	**	0.100	*
CD48	0.050	0.259	0.063	0.160
CD70	− 0.023	0.595	0.135	**
CD80	− 0.086	0.050	0.009	0.842
CD86	− 0.063	0.150	0.053	0.234
CXCL12	0.167	***	0.150	***
CXCR4	0.016	0.712	0.077	0.084
ENTPD1	0.101	*	0.175	***
HHLA2	− 0.017	0.698	− 0.027	0.541
ICOS	− 0.120	**	0.010	0.831
ICOSLG	0.106	*	0.030	0.499
IL2RA	− 0.137	**	0.003	0.941
IL6	− 0.160	***	− 0.031	0.491
IL6R	0.124	**	0.004	0.922
KLRC1	− 0.181	***	0.026	0.554
KLRK1	− 0.113	*	− 0.027	0.546
LTA	− 0.021	0.634	0.028	0.525
MICB	− 0.165	***	0.041	0.362
NT5E	0.012	0.778	− 0.027	0.549
PVR	− 0.263	***	− 0.022	0.621
RAET1E	− 0.175	***	− 0.089	*
TMEM173	0.158	***	0.084	0.061
TMIGD2	− 0.104	*	0.033	0.458
TNFRSF13B	0.129	**	0.014	0.756
TNFRSF13C	0.068	0.125	− 0.006	0.899
TNFRSF14	0.061	0.167	0.159	***
TNFRSF17	0.126	**	0.103	*
TNFRSF18	− 0.018	0.685	− 0.047	0.294
TNFRSF25	0.045	0.311	− 0.064	0.151
TNFRSF4	0.032	0.467	0.042	0.343
TNFRSF8	− 0.062	0.157	0.090	*
TNFRSF9	− 0.141	**	0.065	0.147
TNFSF13	0.089	*	0.045	0.320
TNFSF13B	− 0.041	0.348	0.073	0.101
TNFSF14	− 0.039	0.377	− 0.019	0.669
TNFSF15	0.072	0.103	0.028	0.525
TNFSF18	NA	NA	− 0.154	***
TNFSF4	− 0.090	*	0.099	*
TNFSF9	− 0.143	**	− 0.102	*
ULBP1	0.029	0.511	0.067	0.632

Subsequent analysis of immunomodulators with prognosis of NSCLC patients using Kaplan–Meier plotter displayed that high expression of four immunostimulators (TMEM173, HR = 0.58, *p* = 9.6e−06; IL6R, HR = 0.36, *p* = 5.0e−15; ICOS, HR = 0.49, *p* = 5.4e−09; and ENTPD1, HR = 0.62, *p* = 1.6e−04) and one immunoinhibitor (CTLA4, HR = 0.57, p = 5.6e−06) were related to good prognosis of LUAD patients (Fig. 4G). In contrast, high expression of seven immunostimulators (TNFSF13, HR = 1.89, *p* = 1.3e−07; CD276, HR = 1.74, *p* = 7.2e−06; IL6, HR = 1.69, *p* = 9.9e−06; CD27, HR = 1.38, *p* = 6.7e−03; PVR, HR = 1.54, *p* = 5.0e−04; TNFSF9, HR = 1.5, *p* = 6.5e−04; TMIGD2, HR = 1.71, *p* = 1.3e−05) and one immunoinhibitor (LAG3, HR = 1.34, *p* = 1.3e−02) were associated with poor prognosis of LUAD patients (Fig. 4H). Of these immunomodulators, a high expression of two immunostimulators (CD27, HR = 0.75, *p* = 0.018, and TNFRSF17 (also BCMA), HR = 0.68, *p* = 0.0015) were found to be related with a good prognosis of LUSC patients (Fig. 4I).

## Discussion

CTSF is unique in human cathepsins because of having an extended N-terminal anterior region containing a cystatin domain. Prior studies demonstrated that CTSF may contribute to the progression of gastric cancer, pediatric brain tumors, breast cancer, and lymphoma/leukemia as a suppressor gene [25–28]. Presently, the role of CTSF in NSCLC is still unclear. In the study, the Bioinformatics assessment showed that CTSF might also function as a tumor suppressor gene in NSCLC via regulating immune responses.

Exploration of CTSF expression in TIMER and TCGA datasets showed that CTSF was downregulated in lung cancer tissues related to that of normal lung tissues. Analysis of CTSF expression with clinical characteristics of LUAD patients demonstrated that CTSF expression decreased with stage, metastasis and smoking. High expressed CTSF was significantly correlated with better OS and FS in LUAD patients and better FS in LUSC patients, indicating that CTSF might be a tumor suppressor gene both in LUAD and LUSC, similar to its function in gastric cancer, brain tumors and lymphoma/leukemia. In gastric cancer, downregulated CTSF was found to induce proliferation and inhibit apoptosis of gastric cancer cells [25]. Conversely, progression of gastric cancer was inhibited by upregulated CTSF promoted by LINC00982 binding to transcription factor HEY1 [26]. In brain tumors, CTSF was shown to be lower in ependymoma, glioblastoma, and medulloblastoma compared to normal brain [27]. CTSF knockdown was reported to promote lymphoma/leukemia development when PUMA and p21 were absent [28]. Moreover, CTSF was shown to be one of genes encoding components of the degradome which were reprogramed in acquired resistance to metformin in breast cancer cells [29].

Exploration of CTSF as a tumor suppressor gene in NSCLC by constructing gene–gene interaction network and PPI network demonstrated that besides potential functions of cysteine-type peptidase activity, cysteine-type endopeptidase activity, lysosomal lumen, and vacuolar lumen, CTSF also functions in antigen processing and presentation of exogenous peptide antigen via MHC class II, antigen processing and presentation, and adaptive immune response. The findings were consistent with that of IHC staining of CTSF in normal lung tissues and tumor tissues of NSCLC, which showed that medium staining of CTSF was observed in macrophage cells rather than alveolar cells in normal lung tissues or tumor tissues. Notably, CTSF straining was shown to be stronger in macrophages and other infiltrating immune cells than that of tumor cells in cancerous tissues of NSCLC. Additionally, among the ten proteins interacted with CTSF, nine proteins (HLA-DQA1, HLA-DPA1, HLA-DRA, HLA-DQA2, HLA-DRB1, HLA-DQB1, HLA-DRB5, HLA-DQB2 and HLA-DPB1) are the different subunits of HLA class II, which plays a central role in immune system by presenting peptides derived from extracellular proteins [30]. The other one CTSF-interacted protein CD74 is associates with MHC and also regulates antigen presentation for immune response [31]. The findings mentioned above strongly suggested that CTSF might function as a tumor suppressor gene via contributing to antigen presentation for immune response of NSCLC. Subsequent analysis showing CTSF expression was positively related with immune infiltration of B cells, macrophage, DCs, CD8^+^ T cells, CD4^+^ T cells and Neutrophil in LUAD or LUSC provided evidences that CTSF contributed to immune response in NSCLC.

Contribution of CTSF to immune responses of NSCLC was confirmed by expression correlation of CTSF with immune marker sets and immunomodulators in NSCLC. Expression analysis of CTSF with immune marker sets showed that CTSF expression was positively related with that of molecular markers of B cells, Monocytes, TAMs, M1 macrophages, Neutrophils, DCs, Th2 cells, Tfh cells, Th17 cells and Treg cells in LUAD. In LUSC, CTSF expression was positively associated with that of molecular markers of CD8^+^ T cells, general T cells, B cells, Monocytes, TAMs, M1 macrophages, M2 macrophages, Neutrophils, DCs, Th1 cells, Th2 cells, Th17 cells and Treg cells. Moreover, analysis of CTSF-related immunomodulators with prognosis of NSCLC patients further proved that CTSF affected prognosis of NSCLC via regulating immune responses. Survival analysis displayed that high expression of four CTSF-related immunostimulators (TMEM173, IL6R, ICOS and ENTPD1) and one CTSF-related immunoinhibitor (CTLA-4) signified favorable prognosis of LUAD patients. In contrast, high expression of seven CTSF-related immunostimulators (TNFSF13, CD276, IL6, CD27, PVR, TNFSF9, TMIGD2) and one CTSF-related immunoinhibitor (LAG-3) were associated with poor prognosis of LUAD patients. For LUSC patients, high expression of two CTSF-related immunostimulators (CD27 and TNFRSF17) were related with favorable prognosis. CD27 was the only common CTSF-related immunostimulator associated with prognosis of LUAD and LUSC, although its role in LUAD and LUSC was distinctively different, demonstrating that though LUAD and LUSC belong to NSCLC, CTSF-related immunomodulators in the two subtypes of NSCLC are quite different.

Cancer immunotherapy encompasses a number of different treatments aimed at stimulating immune system in order to promote recognition and elimination of tumor cells [32]. In the past decade, immune checkpoints inhibitors (ICIs) have emerged as anticancer agents targeting inhibitory receptors (e.g. CTLA-4, PD-1, LAG-3, TIM-3) and ligands (PD-L1) expressed on T lymphocytes, antigen presenting cells and tumor cells and elicit an anti-tumor response by stimulating immune system and dramatically improved prognosis of many cancer patients including NSCLC patients [33]. Despite being traditionally considered as exhaustion T-cell markers [34, 35], PD-1, LAG-3 and TIM-3 are expressed preferentially in activated tumor infiltrating lymphocytes (TILs). Among these ICIs, CTLA-4 is a CD28 homolog with much higher binding affinity for B7 and was the first immune checkpoint targeted for cancer therapy in clinical practice [36, 37]. Regretfully, the monodrug therapy that blocked CTLA-4 pathway failed in showed benefit in OS in NSCLC patients [38]. Our findings that high expression of CTSF-related CTLA-4 was associated with better OS of LUAD patients might explain the failure to a certain extent. The underlying mechanism is worth investigating.

LAG-3 (also CD223) is a 498-amino acid type I transmembrane protein with high structural homology with CD4 protein and capacity to bind MHC class II molecules [39, 40]. LAG-3 molecule is expressed on CD4^+^ and CD8^+^ T cells, Tregs, B cells and plasmacytoid dendritic cells. LAG-3 signaling plays a negative regulatory role in T helper 1 (Th1) cell activation, proliferation, and cytokine secretion. MHC-II is considered the canonical ligand of LAG-3 [41]. Currently, Relatlimab (BMS-986016), the first commercially available monoclonal antibody directed against LAG-3, has been used in more than 20 clinical trials [42]. The trial NCT01968109 is evaluating the efficacy of Relatlimab as a monotherapy or in combination with Nivolumab (an anti-PD-1 antibody) in advanced solid tumors as well as NSCLC. PD-1 is expressed in T/B cells, NK, and MDSCs after their activation. In contrast to anti-CTLA-4 antibodies that fulfill their role in initial activation of T cells, main function of PD-1 is to limit activity of T cells in peripheral tissues [43]. Although the monodrug therapy that inhibited CTLA-4 pathway failed in improving OS in NSCLC patients [38], PD-1/PD-L1 (PD-1 ligand 1) checkpoint inhibitors have shown impressive results that have changed the landscape of NSCLC therapy [44–46]. Notably, association of elevated LAG-3 expression with insensitivity to PD-1 axis blockade suggested independence of these immune evasion pathways [47]. In the study, increased expression of CTSF-related LAG-3 with poor prognosis of LUAD patients while no association of CTSF-related PD-1/PD-L1 with prognosis of LUAD patients further demonstrated that the two immune evasion pathways are independent to each other. LAG-3 inhibitors might benefit LUAD patients independent to PD-1/PD-L1 expression.

CD27 (also TNFRSF7) is a member of the tumor necrosis factor receptor superfamily physiologically expressed on CD4^+^ and CD8^+^ T cells, NK cells and thymocytes [48, 49]. By binding to its natural ligand CD70, CD27 signaling enhances T-cell proliferation and differentiation to effector and memory T cells. After CD27 agonistic antibody varlilumab showed promising preclinical efficacy in haematological as well as solid cancers [50–52], varlilumab (also CDX-1127, 1F5), a human monoclonal antibody (mAb) directed at CD27, has entered clinical trials both in haematological cancers and advanced solid tumors (NCT01460134 and NCT02335918) [53–55]. Presently, the clinical trial results are only available for colorectal and ovarian cancer showing that 10% (5/49) of ovarian cancer patients achieved a partial response (PR) and 39% (19/49) a stable disease (SD) [55]. Our findings that the increased expression of CTSF-related CD27 with poor prognosis of LUAD patients while related with favorable prognosis of LUSC suggested that CTSF-related CD27 may play different roles in LUAD and LUSC and hinted that expression level of CTSF and subtype of NSCLC are the two key factors to predict the efficacy of CD27 monoclonal antibody for patients with NSCLC. CD27 monoclonal antibody might benefit patients with LUAD rather than LUSC.

TNFSF13 is a proliferation-inducing ligand playing an important role in B cell development [56]. The clinical significance of TNFSF13 in several cancers was previously analyzed such as NSCLC, breast cancer, leukemia, and other tumor types. In NSCLC, TNFSF13 is shown to be an independent prognostic factor in the 5-year overall survival rate [57]. In acute myeloid leukemia (AML), TNFSF13 is considered as a positive regulator of AML-initiating cells [58]. In triple-negative breast cancer (TNBC), the upregulated TNFSF13 is found to be correlated with a poor response to chemotherapy, suggesting that TNFSF13 could be a predictive biomarker for patients receiving chemotherapy [59]. In the study, CTSF-related TNFSF13 was shown to be related with poor prognosis of LUAD patients. The value of monoclonal antibody targeting TNFSF13 are worth exploring in clinic.

Human cysteine proteinases include more than 10 members [8]. Among these CTSs, several cysteine proteinases have been reported to be associated with progression of cancers by regulating immune response although their functions are entirely different in different cancers. For example, elevated CTSB is associated with increased immune cell infiltration of tumor-associated B cells and mast cells, and facilitates progression and metastasis of PymT-induced mammary carcinomas [60]. CTSS regulates antigen processing and CD4 and CD8 T cell-mediated immune responses. Loss of CTSS activity reduces lymphoma growth by limiting communication with CD4 T follicular helper cells while inducing antigen diversification and activation of CD8 T cells [61]. In colorectal cancer, CTSL is reported to be one of the genes involved in immunosuppression [62]. Increased levels of enzymatically active CTSC contributes to squamous cell carcinoma growth via regulating infiltrating immune cells in neoplastic skin, development of angiogenic vasculature, and squamous cell carcinoma growth [63]. In Endometrial cancer, CTSW is found to be one of the genes correlated positively with tumor infiltration levels of B cells, CD8^+^ T cells, CD4^+^ T cells, macrophages, and dendritic cells, indicating that composition of tumor microenvironment affects clinical outcomes of Endometrial cancer patients, and suggesting that it may provide a basis for development of novel prognostic biomarkers and immunotherapies for patients with Endometrial cancer [64]. Presently, CTSF has been shown to likely play vital roles in immune responses via regulating MHC II [17–19]. Thus, our findings that CTSF and CTSF-related immunomodulators were associated with prognosis of NSCLC strongly suggested that CTSF might play an important role in immune response of NSCLC. Classification of immune cells and immunomodulators based on CTSF expression will help to screen the clinically applicable individuals for immunotherapy. Therefore, targeting CTSF might hew out novel therapeutics of NSCLC by regulating immune responses.

Despite some merits of the current study, there were several limitations. First, because all of the points were speculated from the public databases online available using the bioinformatic methods, the experiments in vitro and in vivo are required to validate the relationship between CTSF and immune responses and explore the mechanisms underpinning CTSF-medicated tumor immunity of NSCLC. Second, the appropriate clinical samples are needed to prove the prognostic impact of CTSF and association between CTSF and immune responses in NSCLC. Third, the agonist or recombinant CTSF need to be explored and tested in animal models to provide novel ways for improving the precision immunotherapy of NSCLC in the future.

## Conclusions

Taken together, the study reported for the first time that CTSF may influence prognosis of NSCLC patients via regulating immune responses and might be a novel therapeutic target of NSCLC.

## Supplementary Information

**Additional file 1**. **Table S1.** Expression of CTSF in human cancer cell lines.

**Additional file 2**. **Figure S1.** (**A**, **B**) Co-expressed genes with CTSF identified in LUAD (**A**) and LUSC (**B**), respectively (Red and green dots represented the positively and negatively correlated genes with CTSF, respectively).

## Data Availability

The datasets used and/or analyzed during the current study are available from the corresponding author on reasonable request.

## Abbreviations

CTSF: Cathepsin F
LUAD: Lung adenocarcinoma
LUSC: Lung squamous cell carcinoma
NSCLC: Non-small cell lung cancer
TCGA: The cancer genome atlas
TIMER: The tumor immune estimation resource
MHC: Major histocompatibility complex
GTE: Genotype-tissue expression
CCLE: Cancer cell line encyclopedia
IHC: Immunohistochemistry
THPA: The human protein atlas
OS: Overall survival
FPS: First-progression survival
PPS: Post-progression survival
HR: Hazard ratio
CIs: Confidence intervals
TMB: Tumor mutational burden
MSI: Microsatellite instability
NGS: Next-generation sequencing
ENCORI: Encyclopedia of RNA interactomes
GO: Gene ontology
KEGG: Kyoto encyclopedia of genes and genomes
PPI: Protein–protein interaction
DCM: Dilated cardiomyopathy
TNBC: Triple-negative breast cancer
AML: Acute myeloid leukemia
ALK: Anaplastic lymphoma kinase
EGFR: Epidermal growth factor receptor
