# Supplementary material for: Expression signature, prognosis value and immune characteristics of cathepsin F in non-small cell lung cancer identified by bioinformatics assessment
Source: BMC Pulm Med. 2021 Dec 20;21:420. doi: 10.1186/s12890-021-01796-w (PMC8686609; doi:10.1186/s12890-021-01796-w)
Supplement: Supplementary file 1 — Additional file 1. Table S1. Expression of CTSF in human cancer cell lines. [file 12890_2021_1796_MOESM1_ESM.docx]

Additional file 1. Table S1 Expression of CTSF in human cancer cell lines

| Cancer Cell Lines | CTSF expression | |
| --- | --- | --- |
|  | Average | Median |
| Autonomic ganglia (n = 17) | 78.73217647 | 81.855 |
| Biliary tract (n = 7) | 68.34685714 | 55.101 |
| Bone (n = 25) | 85.49824 | 72.204 |
| Breast (n = 58) | 110.3748448 | 98.394 |
| Central nervous system (n = 52) | 85.15448077 | 74.8345 |
| Endometrium (n=27) | 78.30381481 | 69.744 |
| Haematopoietic and lymphoid tissue (n = 178) | 52.25839888 | 45.491 |
| Kidney (n = 22) | 58.97368182 | 39.5335 |
| Large intestine (n =5 7) | 51.28087719 | 41.298 |
| Liver (n = 27) | 75.41588889 | 74.595 |
| Lung (n = 174) | 84.53593103 | 68.405 |
| Oesophagus (n = 24) | 77.57966667 | 49.597 |
| Ovary (n = 50) | 89.66528 | 78.927 |
| Pancreas (n = 44) | 62.48968182 | 52.6535 |
| Pleura (n = 10) | 58.6335 | 39.6485 |
| Prostate (n = 7) | 120.6338571 | 107.262 |
| Salivary gland (n = 2) | 39.44 | 39.44 |
| Skin (n = 60) | 106.71615 | 94.649 |
| Small intestine (n = 1) | 77.225 | 77.225 |
| Soft tissue (n = 20) | 105.77485 | 63.741 |
| Stomach (n = 38) | 72.52681579 | 47.5565 |
| Thyroid (n = 11) | 67.34863636 | 59.096 |
| Upper aerodigestive tract (n = 30) | 70.71296667 | 56.811 |
| Urinary tract (n = 24) | 72.51883333 | 54.657 |
