# Supplementary figures and images for: Expression signature, prognosis value and immune characteristics of cathepsin F in non-small cell lung cancer identified by bioinformatics assessment
Source: BMC Pulm Med. 2021 Dec 20;21:420. doi: 10.1186/s12890-021-01796-w (PMC8686609; doi:10.1186/s12890-021-01796-w)

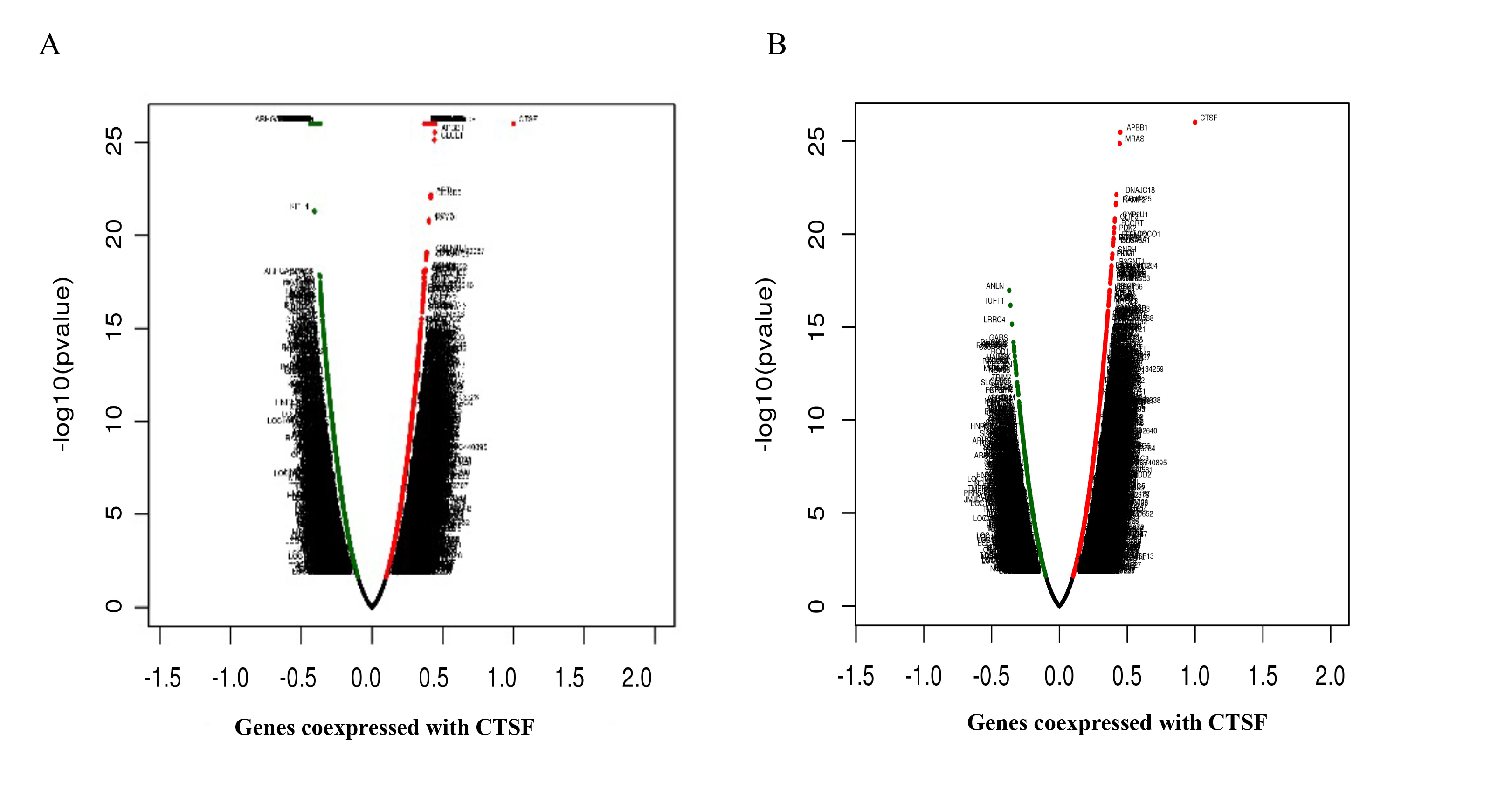

Supplement: Supplementary file 2 — Additional file 2. Figure S1. (A, B) Co-expressed genes with CTSF identified in LUAD (A) and LUSC (B), respectively (Red and green dots represented the positively and negatively correlated genes with CTSF, respectively). [file 12890_2021_1796_MOESM2_ESM.png]
